# Comprehensive survey of transposon *mPing* insertion sites and transcriptome analysis for identifying candidate genes controlling high protein content of rice

**DOI:** 10.3389/fpls.2022.969582

**Published:** 2022-09-02

**Authors:** Yuki Monden, Hirona Tanaka, Ryota Funakoshi, Seiya Sunayama, Kiyotaka Yabe, Eri Kimoto, Kentaro Matsumiya, Takanori Yoshikawa

**Affiliations:** ^1^Graduate School of Environmental and Life Science, Okayama University, Okayama, Japan; ^2^Faculty of Agriculture, Okayama University, Okayama, Japan; ^3^Faculty of Agriculture, Kyoto University, Kyoto, Japan; ^4^Graduate School of Agriculture, Kyoto University, Kyoto, Japan

**Keywords:** amplicon sequencing, protein, rice, RNA-seq, starch, transposon

## Abstract

Rice is the most important crop species in the world, being staple food of more than 80% of people in Asia. About 80% of rice grain is composed of carbohydrates (starch), with its protein content as low as 7–8%. Therefore, increasing the protein content of rice offers way to create a stable protein source that contributes to improving malnutrition and health problems worldwide. We detected two rice lines harboring a significantly higher protein content (namely, HP5-7 and HP7-5) in the EG4 population. The EG4 strain of rice is a unique material in that the transposon *mPing* has high transpositional activity and high copy numbers under natural conditions. Other research indicated that *mPing* is abundant in the gene-rich euchromatic regions, suggesting that *mPing* amplification should create new allelic variants, novel regulatory networks, and phenotypic changes in the EG4 population. Here, we aimed to identify the candidate genes and/or *mPing* insertion sites causing high protein content by comprehensively identifying the *mPing* insertion sites and carrying out an RNA-seq-based transcriptome analysis. By utilizing the next-generation sequencing (NGS)-based methods, *ca.* 570 *mPing* insertion sites were identified per line in the EG4 population. Our results also indicated that *mPing* apparently has a preference for inserting itself in the region near a gene, with 38 genes in total found to contain the *mPing* insertion in the HP lines, of which 21 and 17 genes were specific to HP5-7 and HP7-5, respectively. Transcriptome analysis revealed that most of the genes related to protein synthesis (encoding glutelin, prolamin, and globulin) were up-regulated in HP lines relative to the control line. Interestingly, the differentially expressed gene (DEG) analysis revealed that the expression levels of many genes related to photosynthesis decreased in both HP lines; this suggests the amount of starch may have decreased, indirectly contributing to the increased protein content. The high-protein lines studied here are expected to contribute to the development of high protein-content rice by introducing valuable phenotypic traits such as high and stable yield, disease resistance, and abundant nutrients.

## Introduction

The United Nations expects the world population to reach 9.6 billion by 2050. The current world population is 7.3 billion and its demand for protein is 202 million tons, but this is predicted to increase to 267–286 million tons in 2050 (Henchion et al., 2017). Protein is a polymer of amino acids and an indispensable component of body tissues, enzymes, hormones, etc., as well as a substantial source of essential nutrients and energy. Rice is cultivated worldwide, and 60% of the world’s population depends upon it as a staple food, with more than 80% of people in Asia eating it (Kawakatsu et al., 2008). Rice also supplies 21% of the world’s caloric intake; *ca.* 80% of rice consists of carbohydrates (starch), which is of great value as a staple food, yet its protein content can be as low as 7–8% (Kubota, 2016). Therefore, increasing the protein content of rice would create a stable protein source that can help to overcome malnutrition and health problems not only in Japan but also globally (Chen et al., 2018).

The endosperm of rice is composed of 70–80% starch, 7–10% protein, and 1% lipid (Martin and Fitzgerald, 2002). Rice has four types of seed storage proteins (SSP): albumin, glutelin, prolamin, and globulin. Glutelin is encoded by 15 genes and constitutes 60–80% of the total protein content and is classified into four subfamilies: GluA, GluB, GluC, and GluD (Kawakatsu et al., 2008). Prolamin, encoded by a multigene family of 34 gene copies, makes up 20–30% of total protein and may be categorized as 10 kDa prolamin (RP10), 13 kDa prolamin (RM1, RM2, RM4, and RM9), and 16 kDa prolamin (RP16) according to its molecular weight (Yamagata et al., 1982; Kawakatsu et al., 2008). Globulin is a protein representing 8–10% of total protein; it occurs as two types of polypeptides, 23–27 kDa and 16 kDa, which are structurally homologous to wheat grain glutenin called α-globulin (Ellepola et al., 2006; Kawakatsu et al., 2008). Both albumin and globulin are concentrated in the bran but polishing during the milling process removes a major portion of these proteins (Shewry, 2007). In recent years, enhancing the seed storage proteins to improve rice’s nutritive value has emerged as a key target in rice quality breeding (Jiang et al., 2014). Both the content and composition of protein is crucial to quality of rice grain and its nutritional value (Lin et al., 2005; Chen et al., 2017).

Transposable elements (TEs) are mobile genetic elements in the eukaryotic genome, now recognized as an important source of genome evolution and diversification. TEs are a major component of higher plant genomes, accounting for 35% of the rice genome (Turucotte et al., 2001). TEs may alter the expression of neighboring genes *via* insertion into promoter regions, or disrupt the function of protein-coding genes when inserted into the genes, or even change gene structure by altering its splicing and polyadenylation patterns (Kumar and Bennetzen, 1999; Feschotte et al., 2002; Wessler, 2006; Feschotte and Pritham, 2007; Feschotte, 2008; Butelli et al., 2012). TEs are divided into two classes according to whether their transposition involves either RNA intermediates (Class I) or DNA intermediates (Class II; Kumar and Bennetzen, 1999; Feschotte et al., 2002; Wessler, 2006; Feschotte, 2008). Class I elements are transposed by a “copy and paste” mechanism, which involves the reverse transcription of RNA and the integration of a cDNA fragment. Class II elements are excised and integrated into new genomic locations by a ‘cut and paste’ mechanism. In this respect, Miniature Inverted-repeat Transposable Elements (MITEs) are non-autonomous Class II DNA transposons of small size (< 600 bp) that harbor short terminal inverted repeats (TIRs), capable of attaining high copy numbers in eukaryotic genomes (Feschotte et al., 2002; Feschotte and Pritham, 2007). MITEs have been classified into two superfamilies based on the similarity of their TIRs and their target site duplication (TSD): *Tourist*-like MITEs and *Stowaway*-like MITEs (Feschotte et al., 2002; Feschotte and Pritham, 2007).

*mPing* is the first active MITE through animal and plant genomes, which was discovered independently by three different assays: long-term rice cell culture (Jiang et al., 2003), short-term anther culture (Kikuchi et al., 2003), and plants derived from gamma-irradiated seeds of the rice cultivar (Nakazaki et al., 2013). The *mPing* element is short (430 bp) with 15 bp TIRs and belongs to the *Tourist* family (Jiang et al., 2003). Because *mPing* has no inherent capacity for transposition, the transposase is provided by two related autonomous elements, *Ping* and *Pong*. The autonomous *Ping* and *Pong* elements are members of the *PIF/Harbinger* superfamily that is widespread in both plants and animals (Hancock et al., 2010). Like most members of that superfamily, *Ping* and *Pong* have two open reading frames (ORFs: ORF1 and ORF2), both of which are required for *mPing* transposition from one place to another on the genome (Yang et al., 2007; Hancock et al., 2010). The ORF1 protein contains a conserved *Myb*-like domain whose involvement in DNA binding was hypothesized (Yang et al., 2007; Hancock et al., 2010). The ORF2 encodes the transposase, which contains a putative Asp-Asp-Glu (DDE) motif that is a signature for transposase catalytic centers (Yang et al., 2007; Hancock et al., 2010).

Although *mPing* is clearly an active MITE, its copy numbers are relatively low, with less than 10 copies found in the subspecies *indica* and ~ 50 copies in the subspecies *japonica*, including the sequenced rice genome (Nipponbare; Naito et al., 2006). Another study revealed that *mPing* had amplified to over 1,000 copies in a few *japonica* rice strains (EG4, HEG4, and related landraces), and is still actively transposing and increasing its copy number, by about 20 copies per plant per generation, without radiation (Naito et al., 2006). A comprehensive survey of *mPing* insertion sites in EG4 strain revealed that *mPing* is enriched in euchromatic, gene-rich regions but rarely present in heterochromatic regions (Naito et al., 2009). Considering both the high activity and insertion preference of *mPing* in the EG4 strain, it is reasonable to envision that *mPing* amplification could create new allelic variants and novel regulatory networks, which may generate plants with more phenotypic diversity and/or novel phenotypic traits.

In this study, we investigated the protein content of the EG4 strain known to exhibit high *mPing* activity, and then used next-generation sequencing (NGS) to comprehensively analyze *mPing* insertion sites in the selected EG4 strains with high protein content. Furthermore, an RNA-seq analysis was performed to quantify the expression levels of genes related to protein synthesis. Using the above information, we sought to identify the *mPing* insertion sites and related genes responsible for high protein content of rice.

## Materials and methods

### Plant materials

A total of 396 lines of the EG4 population were grown in the experimental paddy field of Kyoto University, Japan. Genomic DNA was extracted from all plants by using the DNeasy Plant Mini Kit following the manufacturer’s instructions (QIAGEN, Hilden, Germany). The total RNA was extracted from immature seeds on the 7th day after flowering with three biological replicates per line using the RNeasy Plant Mini Kit according to its manufacturer’s instructions (QIAGEN, Hilden, Germany). The extracted RNA was digested with DNase (TAKARA, Shiga, Japan) to remove the remaining gDNA. The yield and quality of the extracted DNA and RNA were confirmed using a NanoDrop 2000 instrument (Thermo Fisher Scientific, Wilmington, DE, United States).

### Measurement of protein content

In this experiment, brown rice was used to quantify the protein content of rice seeds. For practical purposes, it is often desirable to use seeds post-milling to qualify their protein content. However, in this study, brown rice was instead used for two main reasons. First, it is difficult to obtain the minimum amount of brown rice required for the rice milling process. Second, an early study showed a 10% reduction in protein content after milling brown rice, but a strong positive correlation was nonetheless detected between the protein content of brown rice and white rice (Higashi et al., 1974). Hence, it was considered sufficient to quantify the protein content in seeds using brown rice.

In 2013, 396 lines of the EG4 population were cultivated for the primary screening of their protein content. To do this, four seeds per panicle from each line were crushed, and the protein content of their rice powder was measured once by the bicinchoninic acid (BCA) assay method. Based on the measurement results, 25 lines with diverse protein content values were selected and subjected to secondary screening in which total protein content was measured from brown rice by applying the improved Dumas method (Jung et al., 2003). This method burns and reduces the sample at a high temperature, and then measures the amount of nitrogen in the generated nitrogen gas (Jung et al., 2003). This method is quick, requiring just a few minutes to analyze each sample, without the use of any deleterious reagents. Protein content was measured in 700–800 mg of brown rice, three times per line, with SUMIGRAPH^®^ (NC-TRINITY, Sumika Chemical Analysis Service, Ltd, Tokyo, Japan).

### Preparation of the amplicon sequencing library for *mPing* insertion sites

To determine the *mPing* insertion sites in a comprehensive manner, flanking regions of *mPing* insertion sites were amplified by PCR, and the ensuing products were sequenced on an Illumina platform. An amplicon sequencing library was constructed according to previously described methods (Monden et al., 2014, 2015). First, genomic DNA was fragmented using gTUBE (~ 6 kb; Covaris Inc., MA, United States), and forked adaptors were ligated to the fragmented DNA. These forked adaptors were prepared by annealing two different oligos (Forked_Type1 and Forked_Com; Supplementary Table 1). Primary PCR amplification was performed with *mPing*-specific (*mPing_*1st) and adaptor-specific (AP2-Type1) primer combination, which used the adaptor-ligated DNA as the template (Supplementary Table 1). Nested PCR amplification was carried out using the tailed PCR primers (D501-D503 and D701-D712) with primary PCR products serving as the template. The tailed PCR primers contain the P5 or P7 sequence (Illumina) for hybridization on the sequencing flow cell, and several barcodes for multiplex sequencing. Thus, *mPing*-specific primers (i.e., D501–D503) consisted of a P5 sequence, a barcode sequence, and the *mPing* end sequence, while the adapter-specific primers (i.e., D701–D712) consisted of a P7 sequence, a barcode sequence, and an adapter sequence. The primer combinations of each sample can be found in Supplementary Table 2. The ensuing PCR products were size-selected (400–600 bp) on agarose gels and purified with a QIAquick Gel Extraction Kit (QIAGEN, Hilden, Germany). Purified products were then quantified using a Qubit fluorometer (Invitrogen, Carlsbad, CA, United States) and the size selection range was confirmed in an Agilent 2,200 TapeStation system (Agilent, Santa Clara, CA, United States). The MiSeq sequencing library was prepared by pooling equal amounts of purified barcoded products from each line.

### Data analysis

The resulting paired-end reads (150 bp) were analyzed in two ways. The first method followed procedures described in our previous studies (Monden et al., 2014, 2015; Sasai et al., 2019; Hirata et al., 2020), which can detect genome-wide insertion sites of a known TE without any requirement for whole genome sequence information (Monden et al., 2014). The obtained reads were analyzed using Maser, a pipeline execution system of the Cell Innovation Program at the National Institute of Genetics^2^. Adaptor trimming and quality filtering (QV ≥ 30) were performed using cutadapt (Martin, 2011). Filtered reads were trimmed to a specific length that covered most of the sequences. Those reads with ≥10 identical sequences were reduced to a single sequence, in FASTA format, and then clustered using the BLAT self-alignment program (Kent, 2002) under these parameter settings: “-tileSize” = 8, “-minMatch” = 1, “-minScore” = 10, “-repMatch” = −1, and “-oneOff” = 2. This clustering analysis produced many clusters, each corresponding to a separate *mPing* insertion site. An optimal threshold was then set to evaluate the presence or absence of *mPing* insertions: if the number of reads on a given cluster at a specific insertion site comprise < 0.01% of the entire reads for that line, then *mPing* was considered absent from that site. This yielded genotyping information for the presence (1) versus absence (0) of *mPing* insertions in every line.

A second, different analytical method was adopted because the rice reference genome sequence was the first to be deciphered among staple crop species (Eckardt, 2000; Jackson, 2016). Given its subsequent improvements, a highly accurate reference genome sequence of rice is now available (Kawahara et al., 2013; Sakai et al., 2013). The 150-bp paired-end reads were mapped onto the Nipponbare reference genome sequence,^1^ to identify the *mPing* insertion sites. Galaxy/NAAC (Advanced Analysis Center, NARO) was used for the follow-up data analysis. First, low quality reads were removed with Trimmomatic (Bolger et al., 2014). After removing low quality reads, high-quality reads were aligned to the Nipponbare reference genome sequence using BWA (Galaxy Version 1.2.3) software (Li and Durbin, 2009). The number of reads within 500 bp from the start site of each aligned read was counted, using an in-house Perl script, and each site with 50 or more aligned reads were designated a *mPing* insertion site. The MiSeq reads for analyzing the *mPing* insertion sites were deposited under the accession number DDBJ: DRA014320.

### Experimental validation of *mPing* insertion sites

To verify the existence of *mPing* insertions identified by the above data analysis, PCR primers were designed based on the genomic sequence flanking a *mPing* insertion site (Monden et al., 2009; Supplementary Table 3). Each PCR was run in 10-μl reaction volume that contained 5 μl of 2 × Gflex PCR buffer (Mg^2+^, dNTP plus), 0.2 μl of Tks Gflex DNA Polymerase, 1 μl of 10 μM primer (forward and reverse) and 1 μl of genomic DNA (l00 ng/μl). The cycling conditions were as follows: 94°C for 2 min; 35 cycles of 98°C for 10 s, 60°C for 30 s, and 68°C for 30 s; then 68°C for 3 min. Amplified products were visualized using electrophoresis on a 1.5% agarose gel.

### RNA-sequencing

The RNA-sequencing (RNA-seq) library was sequenced using the NovaSeq system (Illumina, San Diego, CA, United States). The paired-end short reads with a read length of 150 bp were analyzed as follows. Adaptor trimming and quality filtering (QV ≥ 30) were performed using Trim Galore,^2^ after which the remaining clean reads were aligned to the Nipponbare reference genome sequence, by using HISAT2 (Kim et al., 2019), and their expression levels were calculated by StringTie (Pertea et al., 2015, 2016). To determine the differentially expressed genes (DEGs), the obtained expression levels were normalized and log-transformed using DESeq2 (Love et al., 2014). Principal component analysis (PCA) was implemented using the “prcomp” function. A biplot graph was visualized with “ggfortify” package in R (Tang et al., 2016). A heatmap was generated by “clustermap” in the seaborn statistical data visualization library in Python (Waskom, 2021). Gene ontology (GO) enrichment analysis was carried out using ShinyGO (Ge et al., 2020). All RNA-seq reads were deposited under the accession number DDBJ: DRA014328.

### Quantitative real-time PCR

The cDNA was synthesized from the total RNA, using the ReverTra Ace^®^ qPCR RT Master Mix (TOYOBO, Osaka, Japan). Next, the qPCR was performed in a Roche LightCycler^®^ 480II system with KOD SYBR^®^ qPCR Mix (TOYOBO, Osaka, Japan). The reaction solution contained 5.0 μl of qPCR Mix, 4.0 μl of cDNA and 1 μl of each primer set (10 μM per forward and reverse). The cycling conditions were as follows: 98°C for 2 min, then 45 cycles of 98°C for 10 s, 60°C for 10 s, and 68°C for 30 s. The expression levels of genes were normalized to the level of constitutive *Actin* expression. All primers used in the qPCR are listed in Supplementary Table 4.

## Results

### Evaluation of protein content in the EG4 rice population

**Figure 1:**
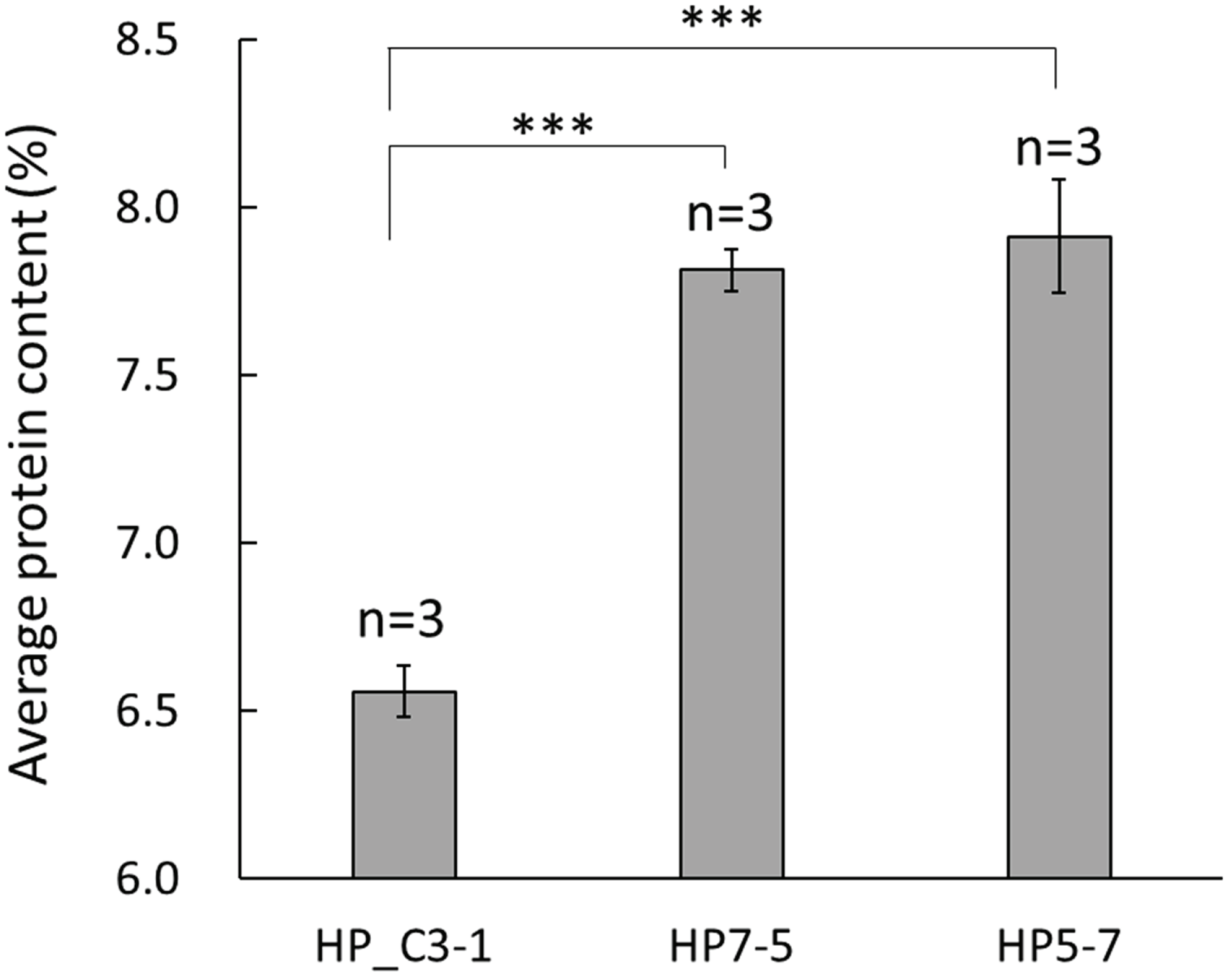
Average protein content in the selected EG4 lines. Protein content was evaluated using three independent plants per line. Data shown are the mean ± SD of three replicates (*n* = 3). A statistically significant difference between the mean values was inferred from Student’s *t*-test (^***^p < 0.001).

In the primary screening step, protein content was measured using the BCA method in 396 lines of the EG4 population. Based on these results, 25 lines with diverse protein content were selected. Secondary screening was performed using the improved Dumas method, and protein content was analyzed in detail. The protein content of the randomly selected EG4 lines was 6.56 to 6.99%, while that of two lines was higher, at 7.91 ± 0.17% and 7.81 ± 0.06%, respectively (Figure 1). Because these two lines had a significantly higher protein content than randomly selected EG4 lines, both (hereon HP5-7 and HP7-5) were selected for use as high-protein lines. In addition, one line (C3-1) featuring normal protein content (6.56 ± 0.08%) was chosen as a control for the subsequent analyses (Figure 1).

### Sequencing and data analysis of *mPing* insertion sites

A total of 16,233,064 paired-end reads of 150 bp were obtained by MiSeq sequencing (min: 533,443, average: 649,323, max: 839,733 reads per line; Supplementary Table 5). After preprocessing, 2,995,488 reads remained overall (Supplementary Table 6). These reads were used for the clustering analysis with the BLAT self-alignment program (Kent, 2002), which suggested 3,268 independent insertion sites in 25 lines (Supplementary Table 6). Next, we determined the genotype (presence or absence) per insertion site and calculated the total number of insertion sites for each line. For the 25 lines, their number of *mPing* insertion sites averaged 573.1, ranging from 495.0 to 655.0 (Supplementary Table 7). The HP5-7 and HP7-5 (high-protein lines) had 589 and 495 *mPing* insertion sites, respectively, while the C3-1 line (control) had 581. These three lines were focused on subsequent analyses.

Given that a highly accurate reference genome sequence is available for rice, we also identified the *mPing* insertion sites in another way, by aligning the MiSeq reads to the Nipponbare reference genome sequence. This yielded 653, 538, and 617 *mPing* insertion sites identified in HP5-7, HP7-5, and C3-1, respectively (Table 1). Comparing the *mPing* insertion sites identified by the clustering-based versus the alignment-based methods in the three lines, 572 (97.1%), 478 (96.6%), and 552 (95.0%) sites were detected in common for HP5-7, HP7-5, and C3-1, respectively (Table 1). Hence, most of the insertion sites (≥ 95%) can be detected by both analytical methods: one which identified insertion sites by clustering the reads based on the sequence similarity, without reliance on the reference genome sequence, and the other doing so by aligning the reads to the rice reference genome sequence. Further, despite having aligned reads < 50 (see “Materials and methods”)—the threshold for determining the presence of absence of insertion—there were some insertion sites where those alignment reads were confirmed. Because these insertion sites were identified by the clustering-based analysis, we considered them actually present. After including these insertion sites, the total number of *mPing* insertion sites per line was estimated to be 580, 485, and 570 in HP5-7, HP7-5, and C3-1, respectively (Table 1).

**Table 1:** The identified *mPing* insertion sites in the three lines of rice.

Subject	C3-1	HP5-7	HP7-5
No. of *mPing* insertion sites based on a clustering analysis	581	589	495
No. of *mPing* insertion sites according to the alignment	617	653	538
No. of common insertion sites	552 (95.0%)	572 (97.1%)	478 (96.6%)
No. of *mPing* insertion sites where reads occurred below the threshold (< 50)	18	8	7
**Total number of *mPing* insertion sites**	**570**	**580**	**485**

To verify the *mPing* insertion sites detected above, an experimental validation was performed using several selected insertion sites. For this, PCR primers were designed based on the flanking sequences of *mPing* insertion sites according to our previously described methodology (Monden et al., 2009). For all insertion sites, the PCR bands of expected size were detected (Supplementary Figure 1). Therefore, the analytical methods in this study were considered highly reliable.

**Figure 2:**
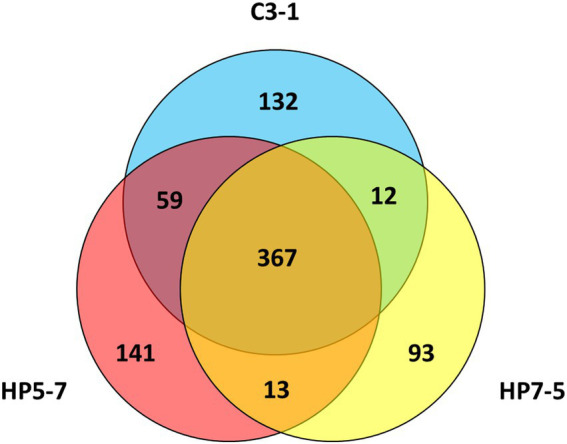
Venn diagram showing the number of *mPing* insertion sites and their overlap among the three lines.

Comparing the *mPing* insertion sites identified in HP5-7, HP7-5, and C3-1, 367 of them (> 60%) were common to these three lines (Figure 2). By contrast, 141, 93, and 132 *mPing* insertion sites were only detected in HP5-7, HP7-5, and C3-1, respectively (Figure 2), which indicated they occurred specifically in each line. These line-specific insertion sites may have arisen very recently.

To gain insight into the effects of *mPing* insertions upon genes, we investigated whether *mPing* insertion sites were located inside genes, near genes (within 3 kb from genes), or in intergenic regions. This revealed 14.0–14.6% of the *mPing* insertions positioned inside genes, 52.8–55.7% of them near genes, and 30.3–32.8% of them situated in intergenic regions (Table 2, Supplementary Table 8). The proportion of inside genes, near genes, and intergenic regions in the rice reference genome was 27.5, 31.0, and 41.5%, respectively (Supplementary Table 9); hence, the frequency of *mPing* insertion is significantly lower inside genes and intergenic regions and higher near genes. In addition, we investigated detailed information on *mPing* insertion sites inside or near genes in the rice genome (Supplementary Table 10). Of the *mPing* insertions located near genes, more *mPing* insertions were detected upstream of the gene as compared to downstream of the gene. Of the *mPing* insertions located inside genes, *mPing* is enriched in introns and 3’ UTR regions. It is possible the *mPing* insertion specific to the HP lines (HP5-7 and HP7-5) might affect their phenotype to increase protein content, so we investigated whether HP line-specific *mPing* insertions occurred inside or near genes. Of the HP5-7 specific insertion sites, 21 insertions were inside a gene and 80 insertions were near a gene (Supplementary Table 11); of the HP7-5 specific insertion sites, 17 insertions were inside a gene and 43 insertions were near a gene (Supplementary Table 12). Therefore, a total of 38 genes were detected that contained an *mPing* insertion unique to HP lines, and we posited they may have lost their function due to *mPing* insertion.

**Table 2:** Distribution of *mPing* insertion sites in the rice genome.

Line	Inside gene		Near gene		Intergenic region		Total
	Number	Proportion (%)	Number	Proportion (%)	Number	Proportion (%)	
C3-1	83	14.6	306	53.7	181	31.8	570
HP5-7	81	14.0	323	55.7	176	30.3	580
HP7-5	70	14.4	256	52.8	159	32.8	485

### Transcriptome analysis

**Figure 3:**
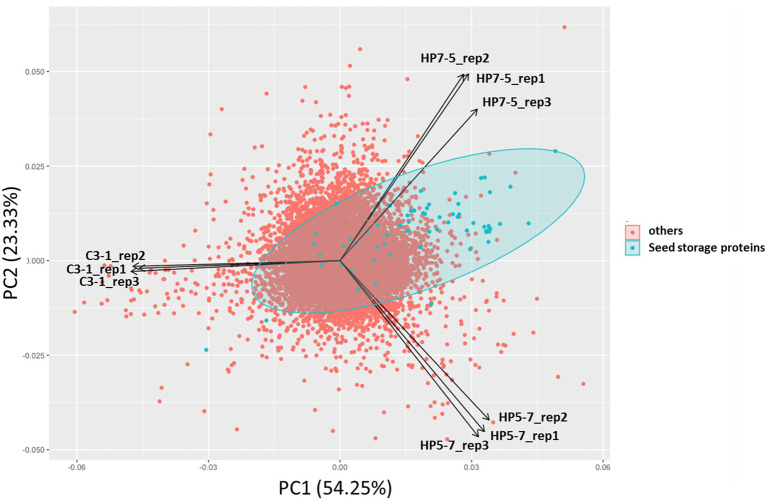
Results of principal component analysis (PCA) based on the expression levels of 37,871 genes. Principal component scores of seed storage protein genes are plotted as green dots.

To investigate how *mPing* insertion affected gene expression, and how the latter is to protein content, RNA-seq-based transcriptome analysis was carried out using immature seeds 7 days post-pollination. In the PCA of expression levels of 37,871 genes, the HP5-7, HP7-5, and C3-1 samples clustered separately in the PCA biplot, while their three replicates per line were close to one another (Figure 3). This indicated the expression patterns of plants belonging to the same line were similar, but differed for plants among the lines. Interestingly, HP5-7 and HP7-5 were distributed in the positive direction of the first principal component (PC1) whereas C3-1 was distributed in the negative direction of PC1 (Figure 3). Accordingly, PC1 was inferred as the axis that reflected protein content of rice. Along PC2, however, HP7-5 and HP5-7 were distributed opposite directions (Figure 3); hence, PC2 was designated an axis that explaining features other than protein content. As expected, principal component scores of seed storage protein genes, when plotted, were strongly biased in the positive direction of PC1 (Figure 3). Therefore, we investigated the expression levels of the genes related to protein synthesis in HP5-7, HP7-5, and C3-1 based on the RNA-seq data.

**Figure 4:**
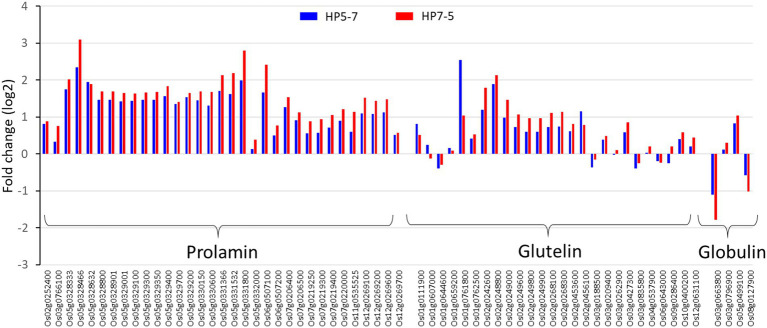
Expression levels of the genes encoding glutelin, prolamin, and globulin, based on the RNA-seq results. The log2[fold-change] value was calculated by comparison with the expression levels of C3-1 (the control).

**Figure 5:**
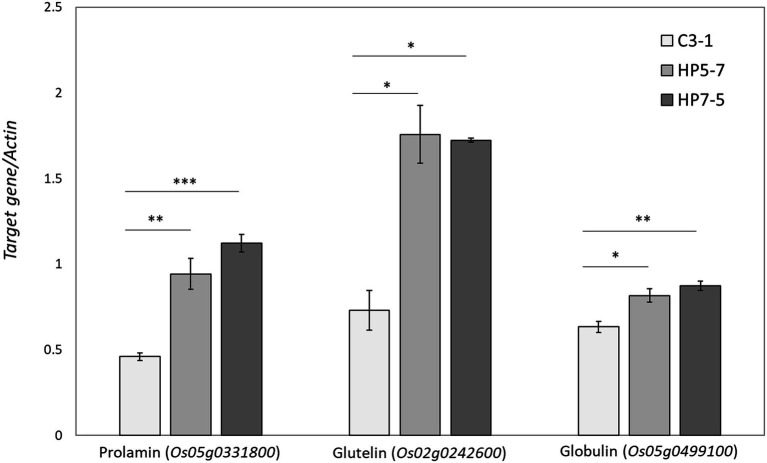
Validation of expression patterns of the selected three genes by qRT-PCR. The expression level of each gene was calculated relative to that of the *Actin g*ene that served as an internal standard. Data shown are the mean ± SD of three replicates. A statistically significant difference between the mean values was inferred from Student’s *t*-test (^*^p < 0.05, ^**^p < 0.01, and ^***^p < 0.001).

The RNA-seq results showed that most of the genes encoding prolamin, glutelin, and globulin were clearly up-regulated in HP5-7 and HP7-5 compared to C3-1 (Figure 4; Supplementary Figure 2). To confirm those results, three genes, respectively, encoding prolamin, glutelin and globulin were randomly selected and subjected to qRT-PCR. Significant differences in expression were confirmed for all genes, and all validated genes had expression patterns similar to those of the RNA-seq data (Figure 5).

**Figure 6:**
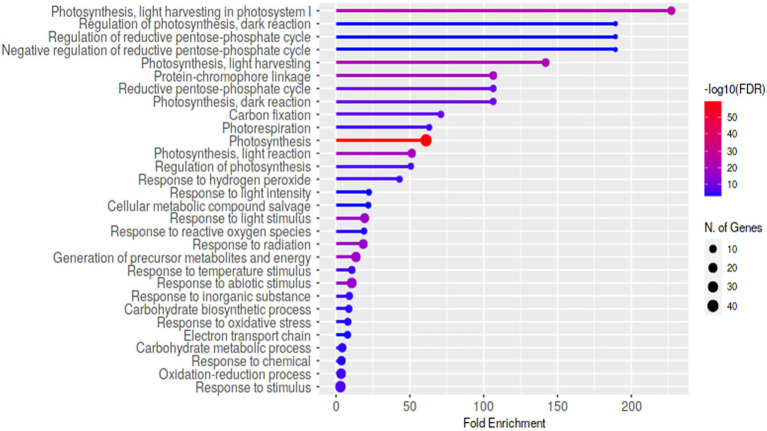
The enriched Gene Ontology (GO) terms of the identified 1,278 DEGs commonly detected in the HP lines. The top-ranked GO terms for the down-regulated DEGs in HP lines compared with C3-1.

Using eXpress and edgeR software tools, differential expression analysis for HP5-7 versus C3-1 revealed 568 up-regulated and 1,910 down-regulated DEGs in HP5-7 (Supplementary Figure 3). Likewise, there were 550 up-regulated and 2,043 down-regulated DEGs in HP7-5 versus C3-1 (Supplementary Figure 3). These results revealed that approximately 80% of DEGs were down-regulated in the HP lines when compared to C3-1. Hierarchical clustering and heatmap expression analyses were then performed using the 1,278 DEGs commonly detected in the HP lines (Supplementary Figure 4). Evidently, expression patterns of these DEGs clearly differed between the C3-1 and both HP lines, whereas those of HP5-7 and HP7-5 were much more similar (Supplementary Figure 4). Compared with C3-1, there were 106 DEGs whose expression was significantly increased in the HP lines (orange cluster in Supplementary Figure 4) and 128 DEGs whose expression was significantly decreased in the HP lines (green cluster in Supplementary Figure 4). We performed a GO enrichment analysis, using ShinyGO, to functionally annotate these DEGs. The top-enriched GO terms for the up-regulated genes in HP lines were “UTP metabolic process,” “UTP biosynthetic process,” “GTP metabolic process,” “GTP biosynthetic process,” and “Guanosine-containing compound biosynthetic process” (Supplementary Figure 5). On the other hand, the top-enriched GO terms for the down-regulated genes in HP lines were “Photosynthesis, light harvesting in photosystem I,” “Regulation of photosynthesis, dark reduction,” “Regulation of reductive pentose-phosphate cycle,” “Negative regulation of reductive pentose-phosphate cycle,” and “Photosynthesis, light harvesting” (Figure 6). This indicated a tendency for decreased expression levels of photosynthesis-related genes in the HP lines, suggesting that the amount of starch, a major product of photosynthesis, might be reduced in the HP lines.

### Differentially expressed genes and *mPing* insertion sites

Finally, to investigate the relationships between gene expression levels of DEGs and *mPing* insertion sites in the HP lines, we extracted those DEGs containing *mPing* insertions or near them (within 3 kb of a gene). Of the DEGs in HP5-7 and HP7-5, an *mPing* insertion was detected near 12 and 10 DEGs, respectively, and one of these DEGs (*Os3g0758551*) was common to both lines (Supplementary Table 13). In addition, for two DEGs (*Os08g0233900* and *Os08g0260400*), the *mPing* insertion inside the gene were detected in HP7-5 (Supplementary Table 13). Against the number of *mPing* insertions near or inside genes (404 and 326 *mPing* insertions in HP5-7 and HP7-5, respectively; Table 2), only 23 DEGs have *mPing* insertions near or inside them. Those results indicated that most of the *mPing* insertions did not affect the expression levels of neighboring genes. Intriguingly, the expression levels of most DEGs with or near an *mPing* insertion were down-regulated, with only two DEGs (*Os10g0532150* and *Os04g0415100*) near an *mPing* insertion found up-regulated (Supplementary Table 13). This suggested *mPing* insertions are more likely to reduce the expression levels of neighboring genes when affecting gene expression. The gene annotations of these DEGs were examined carefully; unfortunately, among them no candidate gene governing the high protein content of HP lines was found.

## Discussion

In this study, two high protein lines (i.e., HP5-7 and HP7-5) were screened from EG4 rice population, and candidate genes and/or *mPing* insertion sites related to high protein content were explored by genome-wide *mPing* insertion sites and an RNA-seq based transcriptome analysis. *mPing* has high transpositional activity and high copy numbers in EG4 and related rice strains under natural conditions (Naito et al., 2009). Considering that *mPing* tends to be inserted into gene-rich euchromatic regions, it is reasonable to suppose that altered gene expression and/or a gene knockout *via mPing* insertion and associated phenotypic changes are likely to occur in the EG4 population. In screening for high-protein content lines from that population based on two independent approaches (primary screening by the BCA assay and secondary screening by the improved Dumas method), we detected two lines (HP5-7 and HP7-5) characterized by high protein content. Utilizing an NGS-based method, a genome-wide analysis of *mPing* insertion sites in EG4 population was completed. These results uncovered approximately 570 *mPing* insertion sites per line for the 25 EG4 lines having variable protein content. The transcriptome results revealed that most of the genes related to storage protein content—encoding glutelin, prolamin, and globulin—were up-regulated in HP lines compared to the control line. We found a total of 38 genes containing an *mPing* insertion that were restricted to the HP lines, consisting of 21 and 17 genes specific to HP5-7 and HP7-5, respectively (Supplementary Tables 11, 12). Focusing on the DEGs, a total of 23 DEGs were detected to have *mPing* insertions inside or near them (Supplementary Table 13). These genes and/or DEGs may be responsible for causing the high protein content in the two selected HP lines. Further research is needed to find genes and/or mutations governing that high protein content.

Rice is a typical self-fertilizing crop, and its genome has been fixed over generations. Generally, the phenotype of these self-fertilizing plants has rarely changed during cultivation, resulting in a uniform population. TEs are considered a pivotal factor for inducing somatic mutations, nucleotide changes, and phenotypic variation in self-fertilizing plants. In the EG4 population, *mPing* is known to be actively transposing and capable of producing approximately 20 new copies per generation without any particular stress (Naito et al., 2006), which should lead to the generation of new allelic mutations and regulatory networks. This study comprehensively investigated the *mPing* insertion sites of several lines from the EG4 population by using the NGS platform. From the rice reference genome sequence, we categorized three regions: inside genes, near genes (within 3 kb from a gene) and intergenic regions, whose corresponding proportions of *mPing* insertion sites in the 12 rice chromosomes were 27.5, 31.0, and 41.5%, respectively (Supplementary Table 9). Yet 14.0–14.6%, 52.8–55.7% and 30.3–32.8% of *mPing* insertion sites were, respectively, located inside genes, near genes, and intergenic regions (Table 2; Supplementary Table 8). Our results suggest *mPing* is more apt to insert itself into the region near a gene. These results are consistent with other studies finding *mPing* enriched in euchromatic, gene-rich regions yet infrequent in heterochromatic regions (Naito et al., 2009, 2014).

Previous studies have analyzed *mPing* insertion sites by a variety of methods. Before the advent of NGS, the copy number of *mPing* was estimated using an experimental method called transposon display (Naito et al., 2006; Takagi et al., 2007). Transposon display, a modified method of amplified fragment length polymorphism (AFLP), has been used to generate and display hundreds of genomic fragments that flank specific transposable elements (Casa et al., 2000). To know the sequence information of a flanking region for the insertion site, the amplified products of interest were excised and purified from polyacrylamide gels. After cloning the purified products, these were individually sequenced piecemeal, using the Sanger method (Naito et al., 2006). But the advent of NGS technology now makes it possible to analyze the *mPing* insertion sites comprehensively at once. Naito et al. (2009) was the first to report on the identification of genome-wide *mPing* insertion sites using high-throughput sequencing technology in EG4 rice and related strains. That paper identified *mPing* insertion sites by amplifying the flanking DNA fragments of *mPing* insertion sites by applying vectorette PCR (Arnold and Hodgson, 1991) and pyrosequencing in the Roche 454 platform. Later, Chen et al. (2019) also characterized *mPing* insertion sites in EG4 and related strains, by using high-throughput short-reads sequencing data. In that paper, the authors analyzed the whole-genome sequencing reads obtained from the Illumina platform using a tool they developed, RelocaTE2 (Chen et al., 2017). RelocaTE2 detects the insertion sites of a known TE using resequencing data, by searching junction reads which contain parts of the TE sequence and parts of the unique host genomic sequence (Chen et al., 2017). In Chen et al. (2019), the copy number of *mPing* was estimated to be 437 in EG4. By contrast, our study found an average of 573.1 (min: 495, max: 655) *mPing* insertion sites identified in each line derived from the EG4 population (Supplementary Table 7). Therefore, our methods can detect more *mPing* insertion sites than that previous study. Moreover, we applied and compared two different analytical methods, clustering-based and alignment-based, to identify the *mPing* insertion sites. More than 95% of *mPing* insertion sites were identified by both methods in the tested three lines (Table 1). These identified insertion sites were amplified as expected by PCR and verified experimentally, confirming our analytical methods are highly effective and reliable.

In this study, we aimed to identify the causal genes underpinning the high-protein phenotype in the HP lines (HP5-7 and HP7-5). Unfortunately, we could not detect any candidate genes. Nevertheless, most of the genes encoding glutelin, prolamin, and globulin were clearly up-regulated in both HP lines (Figure 4). Accordingly, it seems the expression of multiple genes may contribute to the greater protein content, rather than one major gene *per se* being responsible for increasing the protein content. In addition, given the reduced expression of many genes related to photosynthesis, as inferred from the DEG analysis (Figure 6), the starch content likely decreased in HP5-7 and HP7-5. Photosynthesis is a process whereby plants convert light energy into chemical energy; the former is used to convert water, carbon dioxide, and minerals into oxygen and energy-rich organic compounds. In most green plants, carbohydrates, especially starch and the sugar sucrose, are the direct, major organic products of photosynthesis. In a previous study, nitrogen fertilization reduced the expression of genes related to starch synthesis and decreased the storage starch content, while increasing the expression of genes related to amino acid biosynthesis and increasing the storage protein content, implying a trade-off between protein and starch synthesis (Midorikawa et al., 2014). Therefore, a decreased starch synthesis in the HP lines may have increased protein content. When we quantified the starch content in rice landraces varying in their protein content, a strong negative correlation was clearly confirmed between the starch and protein content (*unpublished data*). Based on the above, we posit that the expression of those genes involved in photosynthesis may indirectly contribute to the improved protein content of rice. In the near future, we plan to further investigate both HP lines, in terms of their starch and protein synthesis, which should point to the network of causal genes responsible for their high protein content. Still, for unknown reasons, many DEGs were down-regulated in the HP lines compared with C3-1 (Supplementary Figure 3). It is interesting that thousands of DEGs were detected, even though HP lines and C3-1 have the same genetic background derived from the EG4 strain. Of the detected DEGs, 0.48% (12/2,478) and 0.39% (10/2,593) of DEGs have an *mPing* insertion within 3 kb in HP5-7 and HP7-5, respectively, which indicates that the vast majority of DEGs were not affected by an *mPing* insertion. Accordingly, researchers should also consider the possibility that other factors besides *mPing* might cause an increase in the protein content of HP lines of rice.

## Conclusion

In this study, we selected two rice lines (HP5-7 and HP7-5) with high protein content from the unique EG4 population in which *mPing* is actively transposing under natural conditions, and analyzed their *mPing* insertion sites and their transcriptome. Given that the *mPing* insertion sites identified by NGS were confirmed experimentally, we consider our analytical methods to be highly effective and reliable. Many of the detected *mPing* insertion sites were positioned near the gene (i.e., within 3 kb), which suggests that *mPing* tends to affect the transcription activity of those genes. Transcriptomics revealed that most genes encoding glutelin, prolamin, and globulin were up-regulated in both HP5-7 and HP7-5 lines. Conversely, many genes had expression levels that were lower in the HP lines than the control C3-1 line, and most of the DEGs near *mPing* insertion sites were also down-regulated. In particular, there tends to be reduced expression of photosynthesis-related genes in the HP lines, which suggests that decreased starch content may contribute to greater protein content. In the future, we plan to identify the causal genes responsible for the high protein content of HP lines, by considering the involvement of genes related to photosynthesis and starch synthesis. Further, we anticipate the high-protein lines detected here could lead to the development of high protein rice cultivars by introducing valuable traits such as high and stable yield, disease resistance, and rich nutrient content.

## Data availability statement

The datasets presented in this study can be found in the DDBJ repository, accession numbers DRA014320 and DRA014328.

## Author contributions

TY and YM conceived and designed the experiments. YM, HT, and TY conducted the experiments and data analysis to identify the *mPing* insertion sites. RF and TY performed the transcriptome experiments and analyzed that data. EK and KM measured the protein content of rice seeds. SS performed the experiments that validated the *mPing* insertion sites and transcriptome analysis. KY cultivated the experiments’ rice plants. YM wrote the manuscript. All authors contributed to the article and approved the submitted version.

## Funding

This work was supported by the Japanese Society for the Promotion of Science (JSPS) [KAKENHI, grant number 22H02311 (to TY)] and by the Japanese Science and Technology (JST) agency [COI grant number JPMJCE1307 (to TY)].

## Conflict of interest

The authors declare that the research was conducted in the absence of any commercial or financial relationships that could be construed as a potential conflict of interest.

## Publisher’s note

All claims expressed in this article are solely those of the authors and do not necessarily represent those of their affiliated organizations, or those of the publisher, the editors and the reviewers. Any product that may be evaluated in this article, or claim that may be made by its manufacturer, is not guaranteed or endorsed by the publisher.

## Footnotes

^1^http://cell-innovation.nig.ac.jp/index_en.html(Accessed July 17, 2019).

^2^ https://rapdb.dna.affrc.go.jp/download/irgsp1.html

^3^ https://www.bioinformatics.babraham.ac.uk/projects/trim_galore/

## References

[ref1] Arnold C. Hodgson I. J. (1991). Vectorette PCR: a novel approach to genomic walking. PCR Methods Appl. 1, 39–42. doi: 10.1101/gr.1.1.39, PMID: 1842919

[ref2] Bolger A. M. Lohse M. Usadel B. (2014). Trimmomatic: a flexible trimmer for Illumina sequence data. Bioinformatics 30, 2114–2120. doi: 10.1093/bioinformatics/btu170, PMID: 24695404 PMC4103590

[ref3] Butelli E. Licciardello C. Zhang Y. Liu J. Mackey S. Bailey P.. (2012). Retrotransposons control fruit-specific, cold-dependent accumulation of anthocyanins in blood oranges. Plant Cell 24, 1242–1255. doi: 10.1105/tpc.111.095232, PMID: 22427337 PMC3336134

[ref4] Casa A. M. Brouwer C. Nagel A. Wang L. Zhang Q. Kresovich S.. (2000). The MITE family *heartbreaker* (*Hbr*): molecular markers in maize. Proc. Natl. Acad. Sci. U. S. A. 97, 10083–10089. doi: 10.1073/pnas.97.18.10083, PMID: 10963671 PMC27704

[ref5] Chen L. Lu L. Benjamin J. Diaz S. Hancock C. N. Jason E. S.. (2019). Tracking the origin of two genetic components associated with transposable element bursts in domesticated rice. Nat. Commun. 10:641. doi: 10.1038/s41467-019-08451-3, PMID: 30733435 PMC6367367

[ref6] Chen P. Shen Z. Ming L. Li Y. Dan W. Lou G.. (2018). Genetic basis of variation in rice seed storage protein (albumin, globulin, prolamin, and glutelin) content revealed by genome-wide association analysis. Front. Plant Sci. 9:612. doi: 10.3389/fpls.2018.00612, PMID: 29868069 PMC5954490

[ref7] Chen J. Wrightsman T. R. Wessler S. R. Stajich J. E. (2017). RelocaTE2: A high resolution transposable element insertion site mapping tool for population resequencing. PeerJ. 5:e2942. doi: 10.7717/peerj.2942, PMID: 28149701 PMC5274521

[ref8] Eckardt N. A. (2000). Sequencing the rice genome. Plant Cell 12, 2011–2017. doi: 10.1105/tpc.12.11.2011, PMID: 11090205 PMC526008

[ref9] Ellepola S. Choi S. Phillips D. Ma C. (2006). Raman spectroscopic study of rice globulin. J. Cereal Sci. 43, 85–93. doi: 10.1016/j.jcs.2005.06.006

[ref01] Feschotte C. (2008). Transposable elements and the evolution of regulatory networks. Nat. Rev. Genet. 9, 397–405. doi: 10.1038/nrg2337 18368054 PMC2596197

[ref10] Feschotte C. Jiang N. Wessler S. R. (2002). Plant transposable elements: where genetics meets genomics. Nat. Rev. Genet. 3, 329–341. doi: 10.1038/nrg793, PMID: 11988759

[ref11] Feschotte C. Pritham E. J. (2007). DNA transposons and the evolution of eukaryotic genomes. Annu. Rev. Genet. 41, 331–368. doi: 10.1146/annurev.genet.40.110405.090448, PMID: 18076328 PMC2167627

[ref12] Ge S. X. Jung D. Yao R. (2020). ShinyGO: A graphical gene-set enrichment tool for animals and plants. Bioinformatics 36, 2628–2629. doi: 10.1093/bioinformatics/btz931, PMID: 31882993 PMC7178415

[ref13] Hancock C. N. Zhang F. Wessler S. R. (2010). Transposition of the *tourist*-MITE *mPing* in yeast: an assay that retains key features of catalysis by the class 2 *PIF/harbinger* superfamily. Mob. DNA 1:5. doi: 10.1186/1759-8753-1-5, PMID: 20226077 PMC2836001

[ref14] Henchion M. Hayes M. Mullen A. M. Fenelon M. Tiwari B. (2017). Future protein supply and demand: strategies and factors influencing a sustainable equilibrium. Foods 6:6. doi: 10.3390/foods6070053, PMID: 28726744 PMC5532560

[ref02] Higashi T. Kushibuchi K. Ito R. (1974). Studies on breeding for high protein rice: I. Protein content of different rice varieties and their relations with some agronomic traits including yield. Japan. J. Breed. 24, 88–96.

[ref15] Hirata C. Waki T. Shimomura K. Wada T. Tanaka S. Ikegami H.. (2020). DNA markers based on retrotransposon insertion polymorphisms can detect short DNA fragments for strawberry cultivar identification. Breed. Sci. 70, 231–240. doi: 10.1270/jsbbs.19116, PMID: 32523405 PMC7272250

[ref16] Jackson S. A. (2016). Rice: the first crop genome. Rice 9:14. doi: 10.1186/s12284-016-0087-4, PMID: 27003180 PMC4803718

[ref17] Jiang N. Bao Z. Zhang X. Hirochika H. Eddy S. R. McCouch S. R.. (2003). An active DNA transposon family in rice. Nature 421, 163–167. doi: 10.1038/nature01214 12520302

[ref18] Jiang C. Cheng Z. Zhang C. Yu T. Zhong Q. Shen J. Q.. (2014). Proteomic analysis of seed storage proteins in wild rice species of the *Oryza* genus. Proteome Sci. 12:51. doi: 10.1186/s12953-014-0051-4, PMID: 25505850 PMC4263040

[ref19] Jung S. Rickert D. A. Deak N. A. Aldin E. D. Recknor J. Johnson L. A.. (2003). Comparison of kjeldahl and dumas methods for determining protein contents of soybean products. J. Amer. Oil Chem. Soc. 80:1169. doi: 10.1007/s11746-003-0837-3

[ref20] Kawahara Y. de la Bastide M. Hamilton J. P. Kanamori H. McCombie W. R. Ouyang S.. (2013). Improvement of the *Oryza sativa* Nipponbare reference genome using next generation sequence and optical map data. Rice 6:4. doi: 10.1186/1939-8433-6-4, PMID: 24280374 PMC5395016

[ref21] Kawakatsu T. Yamamoto M. P. Hirose S. Y. Takaiwa F. (2008). Characterization of a new rice glutelin gene *GluD-1* expressed in the starchy endosperm. J. Exp. Bot. 59, 4233–4245. doi: 10.1093/jxb/ern265, PMID: 18980953 PMC2639022

[ref22] Kent W. J. (2002). BLAT—the BLAST-like alignment tool. Genome Res. 12, 656–664. doi: 10.1101/gr.229202, PMID: 11932250 PMC187518

[ref23] Kikuchi K. Terauchi K. Wada M. Hirano H. Y. (2003). The plant MITE *mPing* is mobilized in anther culture. Nature 421, 167–170. doi: 10.1038/nature01218, PMID: 12520303

[ref24] Kim D. Paggi J. M. Park C. Salzberg S. L. (2019). Graph-based genome alignment and genoty*Ping* with HISAT2 and HISAT-genotype. Nat. Biotechnol. 37, 907–915. doi: 10.1038/s41587-019-0201-4, PMID: 31375807 PMC7605509

[ref25] Kubota M. (2016). Novel physiological functions of rice protein. J. Jpn. Soc. Nutr. Food Sci. 69, 283–288. doi: 10.4327/jsnfs.69.283

[ref26] Kumar A. Bennetzen J. L. (1999). Plant retrotransposons. Annu. Rev. Genet. 33, 479–532. doi: 10.1146/annurev.genet.33.1.479 10690416

[ref27] Li H. Durbin R. (2009). Fast and accurate short read alignment with burrows-wheeler transform. Bioinformatics 25, 1754–1760. doi: 10.1093/bioinformatics/btp324, PMID: 19451168 PMC2705234

[ref28] Lin S. K. Chang M. C. Tsai Y. G. Lur H. S. (2005). Proteomic analysis of the expression of proteins related to rice quality during caryopsis development and the effect of high temperature on expression. Proteomics 5, 2140–2156. doi: 10.1002/pmic.200401105, PMID: 15852341

[ref29] Love M. I. Huber W. Anders S. (2014). Moderated estimation of fold change and dispersion for RNA-seq data with DESeq2. Genome Biol. 15:550. doi: 10.1186/s13059-014-0550-8, PMID: 25516281 PMC4302049

[ref30] Martin M. (2011). Cutadapt removes adapter sequences from high-throughput sequencing reads. EMBnet J. 17, 10. doi: 10.14806/ej.17.1.200

[ref31] Martin M. Fitzgerald M. A. (2002). Proteins in rice grains influence cooking properties. J. Cereal Sci. 36, 285–294. doi: 10.1006/jcrs.2001.0465

[ref32] Midorikawa K. Kuroda M. Terauchi K. Hoshi M. Ikenaga S. Ishimaru Y.. (2014). Additional nitrogen fertilization at heading time of rice down-regulates cellulose synthesis in seed endosperm. PLoS One 9:e98738. doi: 10.1371/journal.pone.0098738, PMID: 24905454 PMC4048278

[ref33] Monden Y. Hara T. Okada Y. Jahana O. Kobayashi A. Tabuchi H.. (2015). Construction of a linkage map based on retrotransposon insertion polymorphisms in sweet potato via high-throughput sequencing. Breed. Sci. 65, 145–153. doi: 10.1270/jsbbs.65.145, PMID: 26069444 PMC4430505

[ref34] Monden Y. Naito K. Okumoto Y. Saito H. Oki N. Tsukiyama T.. (2009). High potential of a transposon *mPing* as a marker system in *japonica* × *japonica* cross in rice. DNA Res. 16, 131–140. doi: 10.1093/dnares/dsp004, PMID: 19270311 PMC2671205

[ref35] Monden Y. Yamamoto A. Shindo A. Tahara M. (2014). Efficient DNA fingerprinting based on the targeted sequencing of active retrotransposon insertion sites using a bench-top high-throughput sequencing platform. DNA Res. 21, 491–498. doi: 10.1093/dnares/dsu015, PMID: 24935865 PMC4195495

[ref36] Naito K. Cho E. Yang G. Campbell M. A. Yano K. Okumoto Y.. (2006). Dramatic amplification of a rice transposable element during recent domestication. Proc. Natl. Acad. Sci. U. S. A. 103, 17620–17625. doi: 10.1073/pnas.0605421103, PMID: 17101970 PMC1693796

[ref03] Naito K. Monden Y. Yasuda K. Saito H. Okumoto Y. (2014). mPing: the bursting transposon. Breed. Sci. 64, 109–114. doi: 10.1270/jsbbs.64.109 25053919 PMC4065317

[ref37] Naito K. Zhang F. Tsukiyama T. Saito H. Hancock C. N. Richardson A. O.. (2009). Unexpected consequences of a sudden and massive transposon amplification on rice gene expression. Nature 461, 1130–1134. doi: 10.1038/nature08479, PMID: 19847266

[ref38] Nakazaki T. Okumoto Y. Horibata A. Yamahira S. Teraishi M. Nishida H.. (2013). Mobilization of a transposon in the rice genome. Nature 421, 170–172. doi: 10.1038/nature01219 12520304

[ref39] Pertea M. Kim D. Pertea G. M. Leek J. T. Salzberg S. L. (2016). Transcript-level expression analysis of RNA-seq experiments with HISAT, StringTie and Ballgown. Nat. Protoc. 11, 1650–1667. doi: 10.1038/nprot.2016.095, PMID: 27560171 PMC5032908

[ref40] Pertea M. Pertea G. M. Antonescu C. M. Chang T. C. Mendell J. T. Salzberg S. L. (2015). StringTie enables improved reconstruction of a transcriptome from RNA-seq reads. Nat. Biotech. 33, 290–295. doi: 10.1038/nbt.3122, PMID: 25690850 PMC4643835

[ref41] Sakai H. Lee S. S. Tanaka T. Numa H. Kim J. Kawahara Y.. (2013). Rice annotation project database (RAP-DB): an integrative and interactive database for rice genomics. Plant Cell Physiol. 54:e6. doi: 10.1093/pcp/pcs183, PMID: 23299411 PMC3583025

[ref42] Sasai R. Tabuchi H. Shirasawa K. Kishimoto K. Sato S. Okada Y.. (2019). Development of molecular markers associated with resistance to *Meloidogyne incognita* by performing quantitative trait locus analysis and genome-wide association study in sweet potato. DNA Res. 26, 399–409. doi: 10.1093/dnares/dsz018, PMID: 31377774 PMC6796513

[ref43] Shewry P. R. (2007). Improving the protein content and composition of cereal grain. J. Cereal Sci. 46, 239–250. doi: 10.1016/j.jcs.2007.06.006

[ref44] Takagi K. Ishikawa N. Maekawa M. Tsugane K. Iida S. (2007). Transposon display for active DNA transposons in rice. Genes Genet. Syst. 82, 109–122. doi: 10.1266/ggs.82.109, PMID: 17507777

[ref45] Tang Y. Horikoshi M. Li W. (2016). Ggfortify: unified Interface to visualize statistical result of popular R packages. R J. 8, 474–485. doi: 10.32614/RJ-2016-060

[ref46] Turucotte K. Srinivsan S. Bureau T. (2001). Survey of transposable elements from rice genomic sequences. Plant J. 25, 169–179. doi: 10.1046/j.1365-313x.2001.00945.x, PMID: 11169193

[ref47] Waskom M. L. (2021). Seaborn: statistical data visualization. J. Open Source Soft. 6:3021. doi: 10.21105/joss.03021

[ref48] Wessler S. R. (2006). Transposable elements and the evolution of eukaryotic genomes. Proc. Natl. Acad. Sci. U. S. A. 103, 17600–17601. doi: 10.1073/pnas.0607612103, PMID: 17101965 PMC1693792

[ref49] Yamagata H. Sugimoto T. Tanaka K. Kasai Z. (1982). Biosynthesis of storage proteins in develo*Ping* rice seeds. Plant Physiol. 70, 1094–1100. doi: 10.1104/pp.70.4.1094, PMID: 16662620 PMC1065832

[ref50] Yang G. Zhang F. Hancock C. N. Wessler S. R. (2007). Transposition of the rice miniature inverted repeat transposable element *mPing* in *Arabidopsis thaliana*. Proc. Natl. Acad. Sci. U. S. A. 104, 10962–10967. doi: 10.1073/pnas.0702080104, PMID: 17578919 PMC1904124

