# Supplementary material for: Comprehensive survey of transposon mPing insertion sites and transcriptome analysis for identifying candidate genes controlling high protein content of rice
Source: Front Plant Sci. 2022 Sep 2;13:969582. doi: 10.3389/fpls.2022.969582 (PMC9479144; doi:10.3389/fpls.2022.969582)
Supplement: Supplementary file 2 [file Data_Sheet_1.DOCX]

Supplementary Material


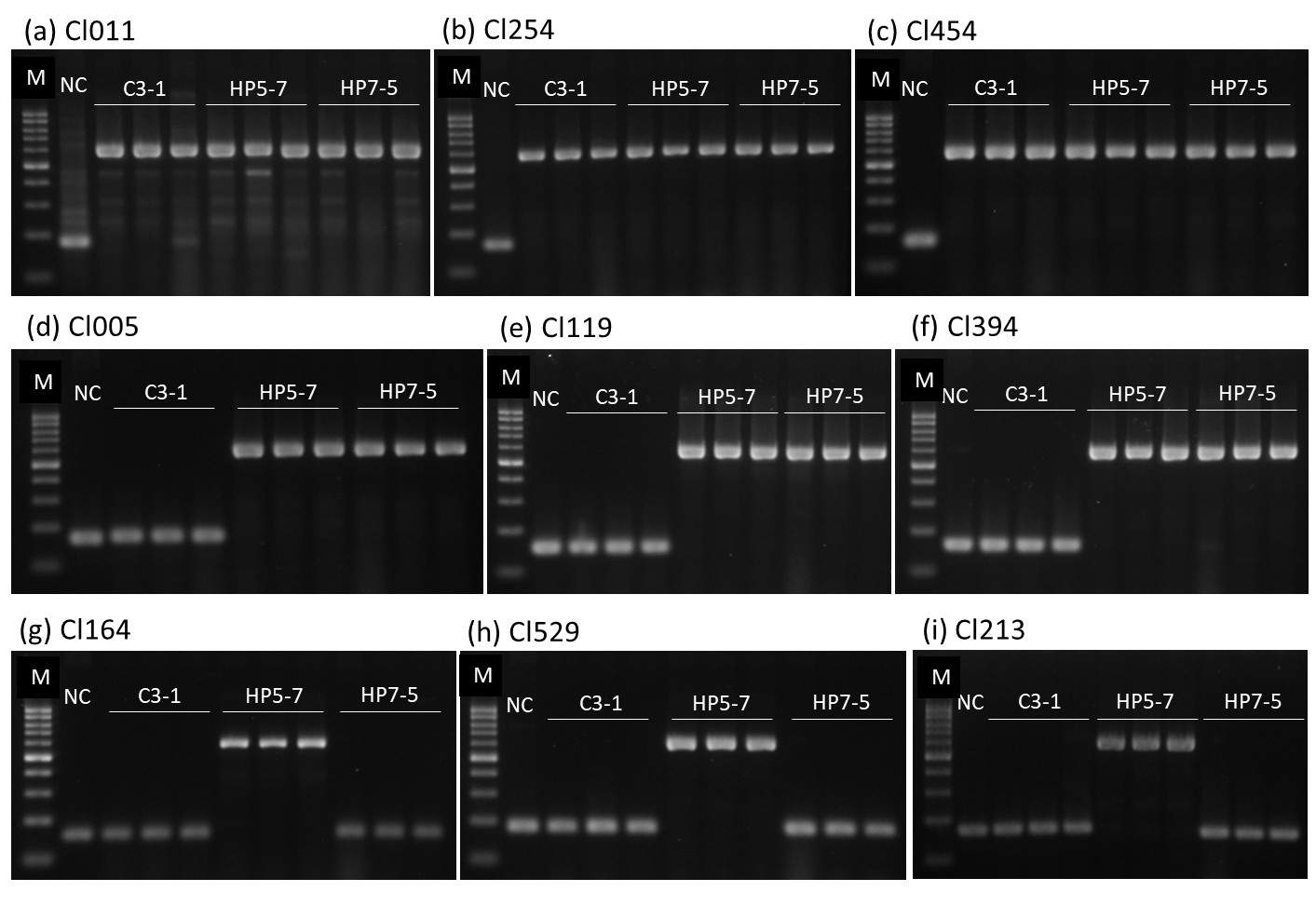


**Supplementary Figure 1.** Experimental validation of *mPing* insertion sites. In those lines having the *mPing* insertion, PCR products that were longer than the length of *mPing* (430 nt) were detected. Three independent plants were used in each line. (a–c) *mPing* insertion present in three lines. (d–f) *mPing* insertion present in HP5-7 and HP7-5. (g–i) *mPing* insertion present in HP5-7. NC: ‘Taichung 65’ rice cultivar. M: 100 bp ladder.


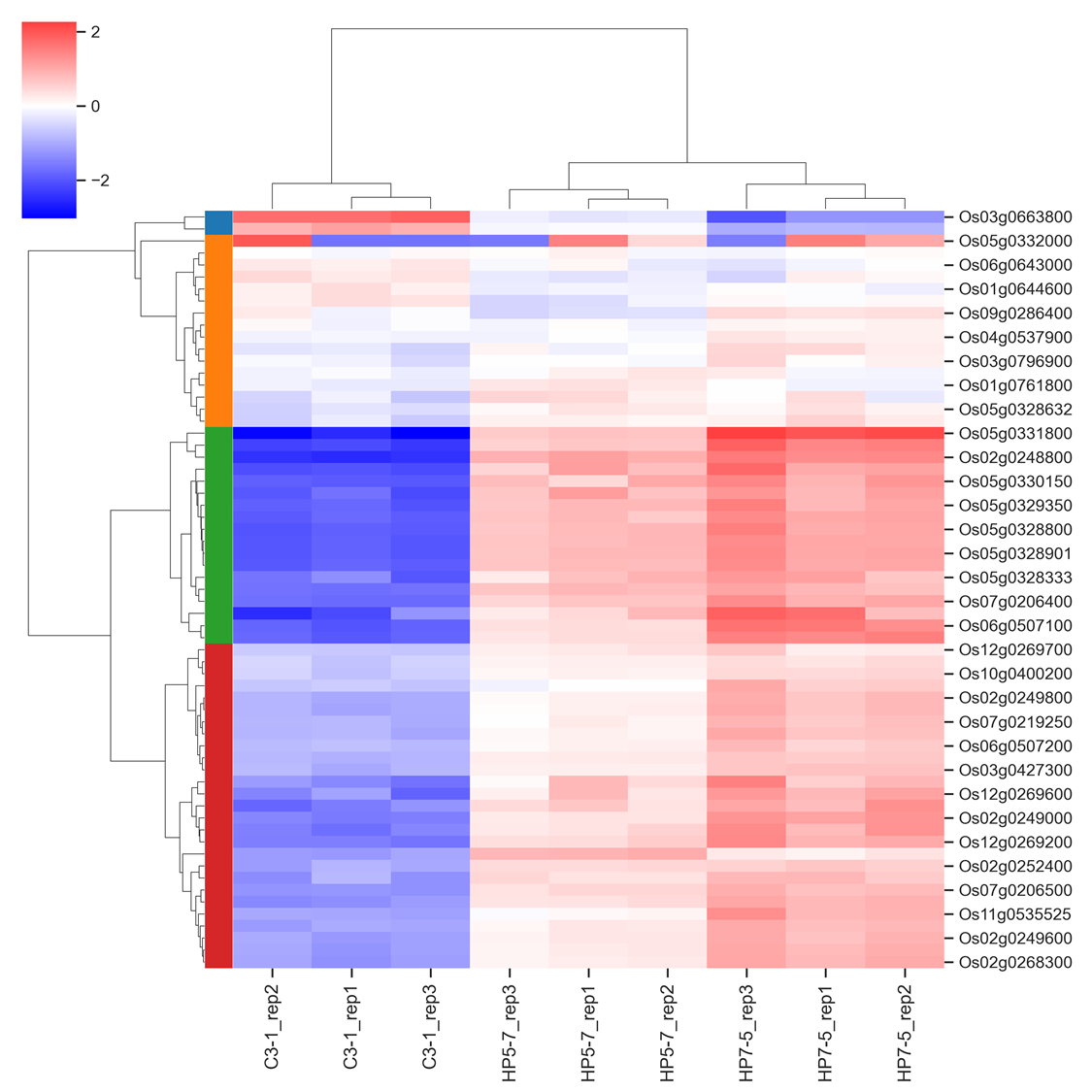


**Supplemental Figure 2.** Hierarchical clustering and expression heatmap based on the genes encoding prolamin, glutelin, and globulin. The gene expression level is represented by the blue (low) to red (high) color gradient.


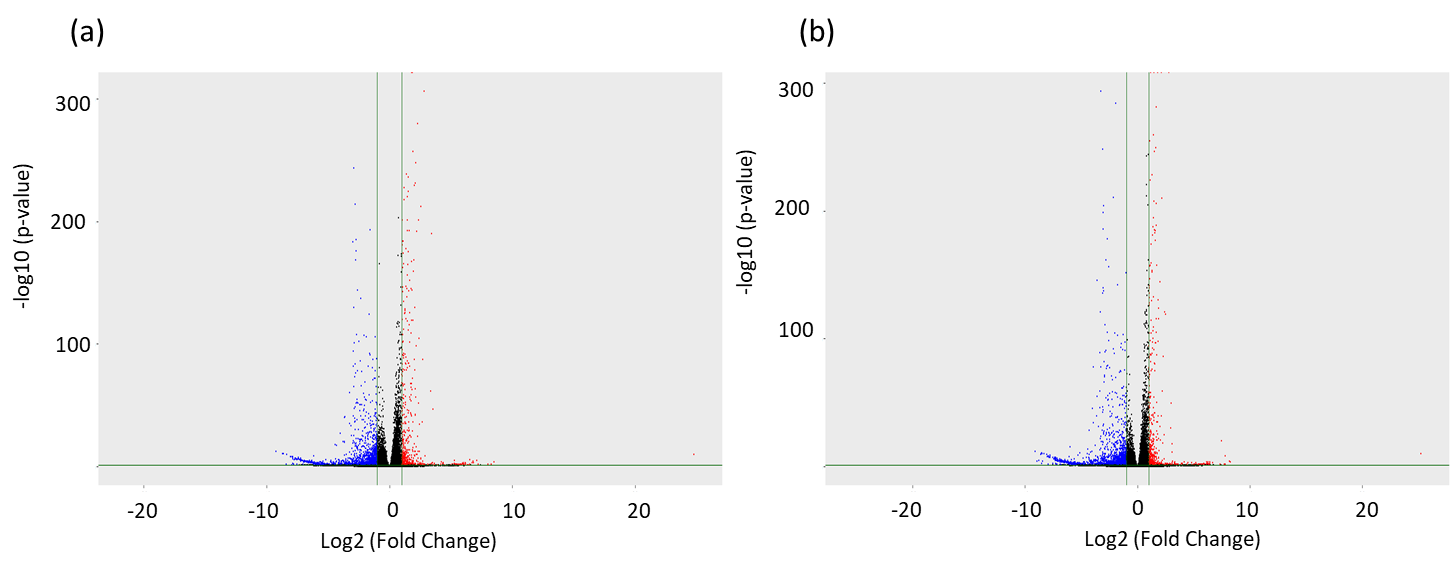


**Supplementary Figure 3.** Volcano plots showing the distribution of DEGs in the HP lines compared with C3-1. Red and blue dots represent those DEGs respectively up-regulated (log2[fold-change] ≥ 1) and down-regulated (log2[fold-change] ≤ –1) with a p-value < 0.05. (a) HP5-7, (b) HP7-5.


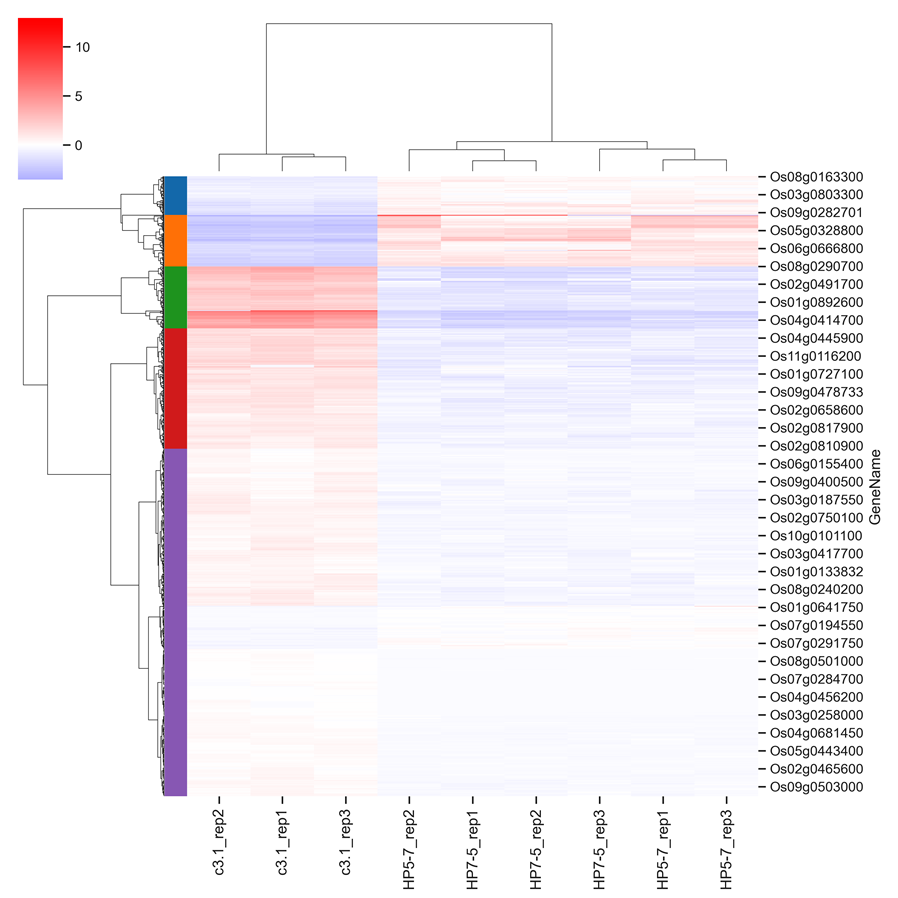


**Supplemental Figure 4.** Hierarchical clustering and expression heatmap based for the 1,278 DEGs. The gene expression level is represented by the blue (low) to red (high) color gradient.


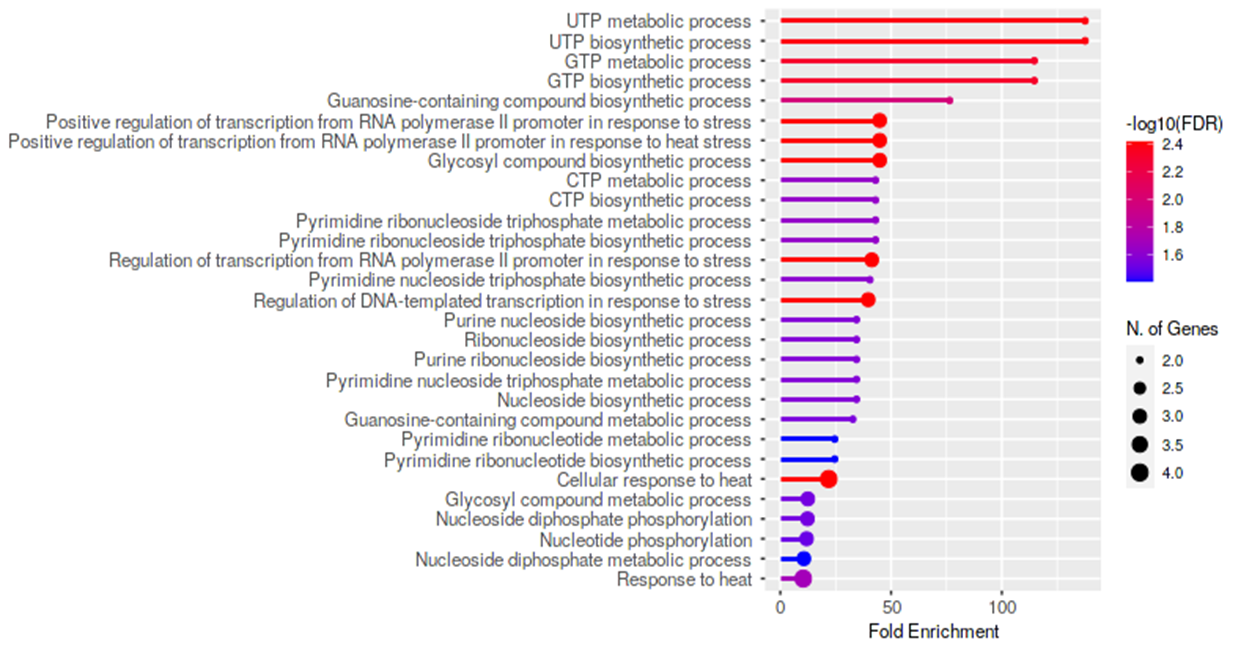


**Supplementary Figure 5.** The enriched Gene Ontology (GO) terms of the identified 1,278 DEGs commonly detected in the HP lines. The top-ranked GO terms for the up-regulated DEGs in HP lines compared with C3-1.
